# The Host–Pathogen Interplay: A Tale of Two Stories within the Cornea and Posterior Segment

**DOI:** 10.3390/microorganisms11082074

**Published:** 2023-08-12

**Authors:** Michael P. Dempsey, Christopher D. Conrady

**Affiliations:** 1 Department of Ophthalmology and Visual Sciences, Truhlsen Eye Center, University of Nebraska Medical Center, Omaha, NE 68105, USA; 2 Department of Pathology and Microbiology, University of Nebraska Medical Center, Omaha, NE 68198, USA

**Keywords:** acute retinal necrosis, innate immunity, ocular immunology, immune privilege, HSV-1, keratitis, endophthalmitis, viral infection

## Abstract

Ocular infectious diseases are an important cause of potentially preventable vision loss and blindness. In the following manuscript, we will review ocular immunology and the pathogenesis of herpesviruses and Pseudomonas aeruginosa infections of the cornea and posterior segment. We will highlight areas of future research and what is currently known to promote bench-to-bedside discoveries to improve clinical outcomes of these debilitating ocular diseases.

## 1. Introduction

The eye is a complex sensory organ directly exposed to the environment and responsible for converting light into electrical signals and eventually images interpreted by the brain. Local tissue changes associated with inflammation and infection can reduce eye function and visual acuity. In fact, an inflammatory event in one eye can affect the function of the clinically “normal” contralateral eye [1]. Complications from ocular infectious diseases are a significant cause of visual impairment, most notably with rates worldwide of 1.4% of total cases of blindness due to corneal opacification from *Chlamydia trachomatis* alone [2]. This results in estimates as high as 5.5 million people who are bilaterally blind from the disease [3]. Within the developed world, herpes simplex viruses are a common pathogen and the leading cause of corneal blindness [4]. As such, infectious diseases of the eye are a significant global burden, whether they be from corneal opacification from trachoma or herpetic keratitis or damage to other structures of the eye such as the retina from *Toxoplasmosis gondii*. Blindness from any cause is more common than previously thought and is estimated to cause a loss in productivity of over USD 400 billion worldwide [5,6] The socioeconomic and psychosocial burden of blindness to a patient, family, and community are substantial with underlying infectious etiologies being potentially treatable, if not completely preventable, common causes of severe vision loss. Unfortunately, our understanding of the host–pathogen interaction is limited within the eye, inhibiting advances in treatment and the prevention of these sight-threatening infections. 

Bacteria and viruses represent the more common causes of ocular infections worldwide and can affect nearly any anatomical part of the eye (Table 1 and Table 2). The overall geographic distribution and common bacterial isolates from ocular infections have been extensively reviewed elsewhere, but *Staphylococcus aureus*, Coagulase negative Staphylococci, *Streptococcus pneumoniae*, and *Pseudomonas aeruginosa* are the leading isolates of bacterial infections of the eye and adnexa (Table 1) [7]. While not specifically evaluated in a large meta-analysis, adenoviruses and herpesviruses are the most routinely encountered viruses within the eye (Table 2) [8]. However, there can be geographic variation in the most common ocular pathogens and rare ocular presentations from unlikely organisms [9,10,11,12,13]. This is most clearly seen with the geographic distribution of the three clades of *Toxoplasmosis gondii* worldwide and the very rare occurrence of *Trypanosome cruzii*-associated retinitis [9,14,15,16]. Within this review, we aim to highlight the known host–pathogen responses by comparing bacterial and viral responses within the cornea and posterior segment and emphasize areas of potential future research to identify therapeutic targets to limit vision loss from these infectious diseases.

## 2. Basics of Ocular Immunology

The eye is a complex sensory organ with parts of it being directly exposed to the surrounding environment with this interface providing an external surface for the establishment of a local microbiome. As such, the host response to infection of the eye is complex as the organ must rid itself of invading environmental pathogens while minimizing immunologically driven pathology from the self or the nonpathogenic microbiota. The combination of the tear film contents (e.g., IgA, Lactoferrin, defensins, and growth factors) and the continuous washing by tears produced by the lacrimal gland serve to remove infectious agents, limit uncontrolled and unregulated inflammatory damage, provide a physical barrier to the surrounding environment, and promote the healing of the ocular surface to reduce the risk of ocular infection [17]. These antimicrobial compounds and barriers alone, however, are insufficient to protect the eye from pathogen invasion. Relatively similar barriers can be found within the retina and posterior segment [18].

Once physical barriers are breached on or within the eye, innate immune mechanisms serve to rapidly identify the pathogen. While the overall immunological response of the eye may slightly differ compared to that of other sites, innate immune sensors are expressed throughout the eye [19,20,21,22,23]. Much like those found in other tissues, Toll-like receptors (TLRs) and other innate sensors recognize conserved pathogen-associated molecular patterns and activate Myeloid differentiation primary response protein (MyD)88-dependent and -independent pathways following complex interactions with other inflammatory receptors [24,25]. NF-κB-related pathways and/or type I interferon (IFN) pathways are preferentially activated by these innate immune sensors [22,26]. Activation of these pathways leads to local innate immune responses including the production of chemokines to facilitate leukocyte trafficking and priming of the more pathogen-specific adaptive immune response [26]. The interplay between many of these pathways will be further highlighted below regarding specific pathogens within distinct areas of the eye.

To further complicate matters, certain parts of the eye are considered “immune privileged” and respond immunologically in ways not seen in other tissues and organs. This is due in part to blood–aqueous and blood–retinal barriers that prohibit the egress of macromolecules into the eye, limiting the exposure of ocular tissue, including local innate immune cells, to circulating pathogens and toxins [27]. Retinal pigment epithelial cells also upregulate T regulatory responses and suppress effector T cell activation [28,29]. This ocular anti-inflammatory milieu with an atypical response known as anterior chamber-associated immune deviation (ACAID) is a phenomenon within the eye in which antigens injected into the anterior chamber induce tolerance whereas the host develops a muted or abolished systemic response to the antigen. While the process is not completely understood, the generation of antigen-specific, antigen-presenting B cells within the spleen following antigen exposure intracamerally results in the development of T regulatory cells and interleukin (IL)-10 production in a relatively immunosuppressive microenvironment within the anterior chamber [30,31,32,33]. γδ T cells and IFN-γ production also appear to be important in this response as their loss does not allow ACAID to develop [34,35]. Subretinal exposure to antigens has been shown to induce a similar response to that induced by ACAID [36]. While different cells, cytokines, and chemokines have all been implicated, ACAID is still not completely understood and it is unclear if this is simply a mouse phenomenon or if it is seen in humans.

The hallmark of immune privilege and immune deviation within the eye is epitomized by the exceptionally high rates of success of corneal transplantation compared to those of other tissues and organs despite the lack of preoperative HLA typing [37]. The success of corneal transplantation appears to be related to the unique nature of the eye in which physical and cellular barriers of the cornea, ACAID-induced by T regulatory cells, and the immunosuppressive microenvironment of the anterior chamber reduce inflammatory reactions, promote immune privilege, and thereby reduce graft failure [37,38,39,40]. However, immune privilege and corneal transplant rejection can be overridden by severing corneal nerves and this rejection process appears to be driven by minor H alloantigens and the loss of regulatory T cells [41,42,43]. Ocular immune privilege is tightly controlled and can be overcome with relatively innocuous changes to eye anatomy.

## 3. The Cornea

### 3.1. Viral Keratitis

Keratitis poses a major global public health concern and can lead to a partial or complete loss of vision due to corneal opacification. Risk factors include specific pathogens (e.g., bacteria, fungi, amebae, and viruses) and forms of exposure (e.g., eye trauma, chemical exposure, ultraviolet exposure, and contact lenses). Viruses are a major cause of infectious blindness worldwide and herpes simplex virus (HSV) type-1 and -2 and varicella zoster virus (VZV) are the most commonly identified viruses [22]. As such, we will focus the viral section of this review on herpesviruses due to space constraints and their visual importance. These viruses are double-stranded DNA viruses that utilize an outer envelope consisting of 16 membrane proteins [44]. These glycoproteins such as gB, gD, gH, and gL represent 12 of the 16 membrane proteins and interact with host cell surface receptors to facilitate the penetration and infection of the cell through either endocytosis or membrane fusion [45,46,47]. Heparan sulfate, through its interaction with gB and/or gC, enables the attachment of the virus to host cells, followed by the binding of viral gD with heparan sulfate or nectin-1 to enable capsid release into the cytoplasm [48]. The capsid is then transported to the nucleus for viral genome replication using host cell DNA polymerases. The new virions produced are released from the cell to infect neighboring cells, and the virus spreads. Primary HSV-1 infection is thought to occur in children or young adults through direct contact with infected mucous membranes. Within the cornea, HSV replication occurs predominately within epithelial cells and results in the clinical development of dendrites and epithelial keratitis. In most cases, this is hypothesized to represent the reactivation of the virus from the trigeminal ganglia. 

HSV-1 infection of any tissue, as with many other viral infections, evokes a strong inflammatory response involving polymorphonuclear leukocytes, macrophages, natural killer cells, and B- and T-cells [49]. While these different cell types are recruited to the site of infection, the following question remains: are they all necessary or is there significant redundancy within the immune response? Further, are all innate responses to all pathogens the same in all tissues? We have previously studied the host immune response to HSV-1 and -2 infection of the cornea. In mouse models of herpetic epithelial keratitis, there are specific immunological responses that occur following viral infection of the corneal epithelium that are similar to those seen in other epithelial tissues such as the rapid induction of antiviral type I IFNs and recruitment of infiltrating leukocytes [50,51]. However, there are distinct differences in the innate immune response to HSV-1 in the cornea compared to that in other commonly affected sites such as the brain. For example, TLR-3 is critical within the central nervous system. A loss of signals emanating from TLR-3 within microglia render mice and children more susceptible to herpetic encephalitis due to the loss of type I IFN production [52,53,54,55]. 

**Figure 1:**
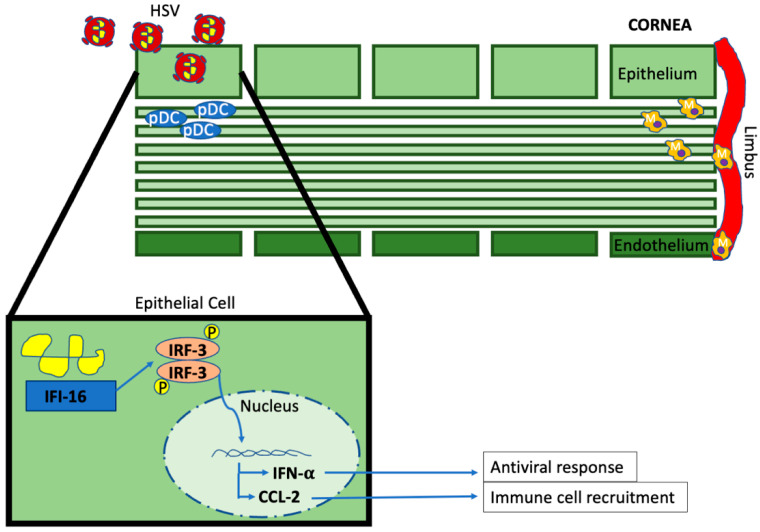
Innate immune response to HSV-1 infection of the cornea. M, macrophages.

In the cornea, we found that the loss of TLR signaling had no effect on viral containment [51]. Rather, the innate response to HSV-1 is driven by a TLR-independent, IFN-inducible protein-16 (p204 mouse homolog)-dependent process to activate type I IFN production and inhibit viral spread through the activation of antiviral proteins such as protein kinase R and 2′,5′-oligoadenylate synthetase (Figure 1) [51,56,57,58]. Additionally, this tightly choregraphed response activates dendritic cells, specific chemokine production, and the recruitment of protective macrophages to the site of infection to further inhibit viral spread [59,60]. Resident plasmacytoid dendritic cells (pDCs), potent producers of type I IFNs, residing in the anterior corneal stroma increase in density following inflammatory changes of the cornea, and their selective depletion results in worse corneal pathology, a reduction in type I IFN production, and HSV-1 dissemination [61]. Antigen-presenting CD11c^+^ dendritic cells traffic to the draining lymph node following exposure to HSV-1 to prime adaptive immunity [62,63,64]. Following this first wave of infiltrating macrophages and the pDC response, type I IFNs induce natural killer (NK) cells and HSV-specific CD8^+^ T cells to undergo maturation and expansion and the leukocytes are then recruited to the site of infection [60,65,66,67,68,69]. These infiltrating cells further inhibit viral proliferation and spread [63]. γδ T cells are also emerging as important recruited leukocytes in HSV defense within the cornea [68]. It is unclear what, if any, other leukocyte populations contribute to viral immunity or pathology in the cornea during this early response. Once the virus has been controlled and establishes latency, typically in the trigeminal ganglia, HSV-specific CD8^+^ T cells are critical in inhibiting reactivation [70,71,72,73,74].

If the earliest steps in this response are inadequate or aberrant, chemokines become dysregulated, immune cell trafficking is altered, and the virus disseminates [56,62,65]. This highly regulated process is best epitomized by comparing wild-type and knock-out mice lacking a functional type I IFN receptor as these mice are unable to respond to type I IFNs. Within these knock out mice, we found that viral loads are significantly higher locally and systemically, the development of encephalitis is rapid, there is a loss of draining lymph node integrity, and there is aberrant chemokine production, specifically a loss of CCL2 and CXCL10 and an overproduction of CXCL1, within the first few days after infection with HSV-1 when compared to wild-type controls [51,53,56,59,62,65]. This resulted in a loss of infiltrating monocytes and the primary source of the antiviral compound nitric oxide [59,75,76,77,78]. The importance of macrophages early in HSV-1 infection of the cornea has been corroborated by others [79,80]. 

Late in the disease’s process, lymphangiogenesis is a common complication of herpetic infections of the cornea. Vessel ingrowth has been shown to be driven by the infected cell protein-4 of HSV-1 which binds directly to the vascular endothelial growth factor (VEGF)-A promoter to induce corneal neovascularization [81,82]. Further, an imbalance between the anti-neovascular soluble VEGF receptor-1 and VEGF develops during infection, promoting neovascularization [83]. Multiple immune cell types including CD4^+^ and CD8^+^ T cells and neutrophils produce VEGF subtypes and increase the risk of corneal graft failure [63,84,85]. The host immune response to the virus, specifically infiltrating antigen-specific and nonspecific CD4^+^ T cells that arrive within 7 days post-infection, has been implicated in the development of corneal opacification during stromal keratitis, another late but common complication of herpetic keratitis [86,87]. In further support of these findings, mice lacking T cells do not develop stromal lesions and the adoptive transfer of CD4^+^ T cells into these same mice restores lesion formation [88,89]. This is supported by the clinical use of topical corticosteroids during herpetic stromal keratitis to quell disease [90]. These topical corticosteroids likely inhibit the function of infiltrating neutrophils as well as other cell types that have been shown to damage the cornea by producing proinflammatory compounds including metalloproteinases and VEGF [91,92]. An intact complement pathway has been shown to be a potent inhibitor of these late complications due to the development of tolerance mediated by iC3b [93,94]. Thus, it is clear that there is a critical antiviral response and also a nonprotective, locally destructive response to HSV-1 infection within the cornea.

### 3.2. Bacterial Keratitis

The pathogenesis and immune reaction to herpetic keratitis contrasts that of bacterial infection of the cornea, or bacterial keratitis (BK). BK is a serious, vision-threatening eye infection that may lead to permanent vision loss due to corneal scarring, even among treated patients, or in rare cases, the development of endophthalmitis [95]. There is a risk of corneal perforation from extreme thinning and extension into surrounding tissues is possible. This ophthalmic infection requires prompt treatment to reduce the development of these ocular complications. Predisposing risk factors for BK include contact lens wear, previous topical steroid use, ocular surface disease (OSD), ocular trauma, previous keratitis, prior surgery, and ongoing corneal disease [96,97,98,99]. 

*Pseudomonas aeruginosa* is the most common causative agent in cases of BK associated with contact lens use [100]. Corneal infections with *P. aeruginosa* often have poor clinical outcomes and can result in long and costly treatments. Due to its ocular importance and representative preclinical models, we will focus our BK discussion on *P. aeruginosa*. Much like many other pathogens, *P. aeruginosa* requires a compromised ocular surface to penetrate and infect the cornea. Contact lens use increases the susceptibility of the cornea to infection due to several factors, mostly by causing a compromised ocular surface. These factors include a relative hypoxic state, ocular trauma, microtrauma to the corneal epithelium, herpetic keratitis, an immunocompromised state, and improper eye hygiene, which are related to or risks amplified by contact lens use [101]. Once *P. aeruginosa* is introduced and/or internalized by a compromised host epithelium, the pathogen is able to invade the cornea [102]. During the infection process, the pathogen exploits its large genome, encoding complex regulatory networks and a wide range of virulence factors, including motility and the secretion of various proteases and toxins [101]. There is increasing evidence that multidrug-resistant infections are associated with a phylogenetic subgroup of *P. aeruginosa* isolates carrying the gene encoding the potent cytotoxin exotoxin U, one of two mutually exclusive exotoxins secreted via the type III secretion system, and may be associated with worse clinical outcomes [103,104,105,106]. The bacterium has developed mechanisms to evade innate immune recognition [107]. Consequently, *P. aeruginosa* has been and will be an important and difficult cause of BK to treat within ophthalmology clinics due to emerging pathogen virulence factors and evasion mechanisms.

**Figure 2:**
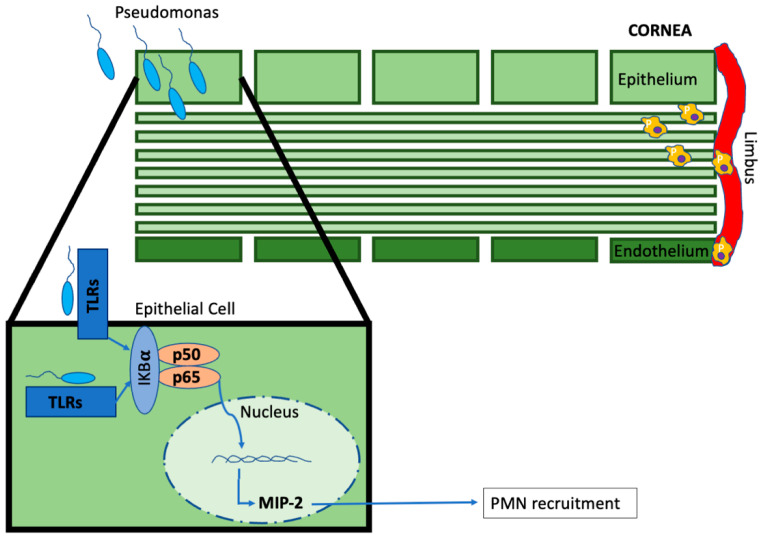
Innate immune response to bacterial infection of the cornea. P, neutrophils.

The host response to *P. aeruginosa* in the cornea is complex. In a mouse model of *P. aeruginosa*-associated keratitis, a loss of signals emanating from the MyD88 adaptor protein resulted in uncontrolled bacterial replication and spread; however, the loss of neutrophil recruitment resulted in a less severe corneal pathology in these same mice [108]. These findings emphasized the role of neutrophils in the cornea to contain the pathogen but at a cost of worsening local tissue destruction. This was supported with the knockdown of TLR-9 expression resulting in higher bacterial loads but fewer infiltrating polymorphonuclear leukocytes/neutrophils (PMNs) and corneal perforations, reduced inflammatory chemokine concentrations, and clearer corneas compared to controls [109]. Interestingly, the loss of TLR-4 in these infections reduced bacterial defense but also led to increased mRNA expression of proinflammatory cytokines, more infiltrating neutrophils, and higher rates of corneal perforation [110]. Thus, TLR-4 and -9 appear to have roles in bacterial containment but diverging roles in the development of a proinflammatory milieu. Pretreatment of the cornea with the TLR-5 agonist, flagellin, a known innate immune sensor of *P. aeruginosa*, through an interferon regulatory factor (IRF)-1-dependent pathway, reduces susceptibility to infection (Figure 2) [111,112]. Taken together, TLR activation within the cornea has protective and destructive implications within the cornea. It is unclear if each of these TLRs regulate specific responses or are redundant, activating similar downstream pathways.

Appropriate numbers of neutrophils appear to be an important leukocyte population in BK due to the production of several antibacterial compounds or responses from these cells [113]. Neutrophil extracellular trap formation (“dead zones”), activation of the transcription factor hypoxia inducible factor-1α, and the production of reactive oxygen species from PMNs inhibit *P. aeruginosa* spread beyond the outermost corneal layers [114,115,116]. PMNS are recruited to the cornea by macrophage inflammatory protein (MIP)-2, and the neutralization of this chemokine resulted in fewer infiltrating PMNs and less corneal damage but no change in bacterial counts (Figure 2) [117] An alarmin, high-mobility group box 1 (HMGB1), induces proinflammatory signals including the production of MIP-2 during these infections and can be downregulated by vasoactive intestinal peptide [118,119]. The production of MIP-2 to recruit is tightly regulated by IL-1 and the prolonged production of IL-1 results in PMN persistence, increased tissue destruction, and corneal perforation [117,120]. The regulation of MIP-2 by IL-1 has been extensively reviewed previously and their role in PMN infiltration during BK is critical in both pathogen clearance and the development of corneal pathology [113]. 

Innate immune activation and the recruitment of early infiltrating immune cells prime the adaptive response following BK. Activated CD4^+^ and CD8^+^ T cells are recruited into the cornea by MIP-1α by day 5 post-infection [121]. Unfortunately, a predominantly Th1 CD4^+^ T cell response in C57Bl/6J mice is associated with much higher rates of corneal perforation and higher bacterial counts than that seen with a Th2-driven response in BALB/c mice [121]. Further, activated T cells have been shown to enhance the recruitment of PMNs to the site of infection and PMN persistence [113,122]. Consequently, a Th1 response appears to be detrimental to the host defense response and local tissue integrity. The precise role of a Th2 response within the cornea to *P. aeruginosa* is not well-elucidated. However, exogenous IL-36α can overcome the Th1 signals in C57Bl/6J mice and drive a Th2 response, and conversely, IL-17 inhibits Th2 cytokine production [123,124]. Similarly to what is observed with viral infections of the cornea, an innate and adaptive immune response to bacterial infection is required to contain the pathogen but can cause local tissue pathology.

## 4. Infections of the Posterior Segment

### 4.1. Viral Retinitis

While rare, herpes viruses can affect the posterior segment as well. HSV-1, -2, and VZV are the most common causes of acute retinal necrosis (ARN), a rapidly progressive infection of the retina leading to severe vision loss and high rates of visual complications in children and adults [125,126,127]. There is broad upregulation of inflammatory markers within the eye in humans with the disease [128,129]. Mouse models of the disease have been created to simulate ARN with the most defined model, the von Szily model, one in which intracameral injection of HSV-1 into BALB/c mice results in anterior chamber inflammation in the injected eye and eventually the development of retinitis in the contralateral eye due to two waves of the virus invading the retina and a delayed hypersensitivity reaction [130,131,132]. With this model, there is broad upregulation of genes and inflammatory compounds within the posterior segment and neutrophils appear to inhibit the development of retinitis [133,134,135]. Tumor necrosis factor (TNF)-α, a proinflammatory cytokine and major regulator of inflammation, is upregulated, and its neutralization reduces retinal pathology [133,136]. Within areas of retinitis, infiltrating T cells, activated Muller cells, retinal pigment epithelial cells, PMNS, and macrophages can be found [133,137]. It is unclear what protective or pathology-causing role these local or infiltrating immune cells and inflammatory chemokines and cytokines have on the pathogenesis of ARN. However, the broad cellular infiltrate supports the broad upregulation of immune pathways and a robust local inflammatory milieu.

While we have learned much about both innate and adaptive immunity from the von Szily model, we felt there were limitations with the model in the study of innate immunity. These limitations include a lack of mouse immune knock-outs on a BALB/c background, the imprecise timing of retinal infection, and the variance from the typical clinical presentation (e.g., preceding inflammation in the contralateral eye is exceedingly rare) [138]. To directly infect the retina and create a novel mouse model more reminiscent of human disease to study innate immunity, our lab began utilizing an adapted mouse model of retinal detachments that had been previously used in rabbits [139,140]. Rather than injecting viscoelastic, our lab injects HSV-1 directly into the subretinal space of C57Bl/6J mice through a focal retinotomy, and after several days, the subretinal bleb resorbs and the mouse develops focal viral retinitis similar to that seen in humans [141]. We have found that much like in other tissues, type I IFNs and downstream antiviral responses are critical in containing the virus as mice lacking a functional type I IFN receptor have worse local tissue destruction, higher viral titers, and dissemination of the virus into the brain leading to mouse demise by day 5 post-infection, which is not seen in wild-type controls [51,56,62,141,142]. While the innate response within these mice and their extreme susceptibility to viral infection of the retina is not completely understood, there appear to be changes in the cell death pathways and chemokines produced compared to those in wild-type mice and controls [141]. While retinal infections with cytomegalovirus appear and progress very differently from how ARN does clinically, cell death pathways are also broadly upregulated and a loss of caspase-1, a mediator of programmed cell death, results in atypical retinal disease [143,144]. In other neuronal tissues, cell death pathways also play an important role in limiting herpetic encephalitis [145]. Within ARN, it is unclear what activation of these pathways is regulating, minimizing, or worsening in the disease’s process. We hypothesize that we will be able to identify potential therapeutic targets within cell death and/or innate immune pathways that can be targeted to reduce local tissue destruction while retaining viral control, improving clinical outcomes with our mouse model. Despite these two mouse models of disease, our understanding of innate and adaptive immunity to HSV-1 within the retina is limited, thus inhibiting advances in other ocular infectious and noninfectious diseases in which the innate immune system has been implicated or is required (e.g., diabetic retinopathy and age-related macular degeneration).

### 4.2. Bacterial Endophthalmitis

While bacterial infections of the retina are rare, bacterial endophthalmitis is one of the most devastating complications of any intraocular procedure, and despite the current strategies for prevention, early identification, and aggressive treatment, substantial vision loss still frequently occurs [12,146,147,148,149,150]. Endophthalmitis can more rarely be associated with blood-borne infections (endogenous), or more commonly, following trauma or surgery to the eye (exogenous). Several bacterial organisms that cause endophthalmitis, such as *Streptococcus pneumonaie*, *P. aeruginosa* or other gram (-) organisms, have been associated with exceptionally poor visual outcomes and higher rates of evisceration or enucleation [12,151,152]. Fortunately, gram (-) organisms are rare and gram (+), coagulase-negative bacteria are the most commonly identified pathogens [148,153]. Due to the higher frequency of gram (+) organisms, we will focus this portion of the review on immunity to bacterial infections of the posterior segment from gram (+) organisms.

Once relatively high doses of *Staphylococcus* are introduced into the posterior segment via trauma or following an intraocular procedure, cell wall components such as peptidoglycan and lipoteichoic acid induce the production of proinflammatory chemokines and cytokines such as IL-6, TNF-α, CXCL-1, and MIP-2, while the bacterial toxins upregulate IL-1β [154,155]. Bacterial loads and inflammatory markers such as IL-1β, TNF-α, and CXCL1 peak within mice within 24 h of inoculation [155]. These inflammatory markers are also elevated in *Staphylococcus epidermidis* and *Streptococcus pneumonia* endophthalmitis very early on, leading to increased vascular permeability [154,155,156]. The early bacterial and inflammatory changes within the eye are associated with a 50% reduction in electroretinogram (ERG) amplitude in mice and the development of a local pathology within 12–24 h of infection that persists until at least 72 h but likely lasts much longer based on poor clinical outcomes [148,155,157]. Various innate immune sensors have been implicated in bacterial defense including TLR-dependent and -independent pathways within virtually all cell types within the retina and retinal pigment epithelium [20,21,158,159]. These sensors are upregulated following infection for unclear reasons but we hypothesize that they could accentuate immunopathology with their subsequent activation [155]. The NLRP3 inflammasome appears to regulate IL-1 production within PMNs and microglia, and the sensors’ dysfunction results in higher bacterial loads and worse retinal tissue damage [21,160]. Mice unable to produce CXCL1 have an attenuated and delayed development of intraocular inflammation and ERG changes despite the lack of differences in bacterial load compared to that of controls at multiple time points [157]. S. aureus produces several toxins that have been shown to induce host tissue changes including within the eye [161,162]. The neutralization of these toxins with nanospores improves retinal function (higher A- and B-wave retention) despite the similar bacterial loads of untreated but infected control mice [163]. Additionally, strains deficient in certain toxins have been shown to be associated with less retinal damage [162]. Taken together, these findings suggest that a higher bacterial burden, toxin production, and enhanced host inflammation are responsible for the development of retinal pathology. Thus, our understanding of immunity and neuropathology to bacterial endophthalmitis is in its infancy but may yield important clues and identify potential therapeutic targets, mitigate retinal pathology, and promote visual rehabilitation. What is readily apparent is that clinical treatments and interventions must be performed early on in the disease’s trajectory as pathological and ERG changes are seen within 12–24 h after infection in mice [155,157].

## 5. Conclusions: The Yin and Yang of Immunity in the Eye

The innate and adaptive immune responses within the eye are important inhibitors of pathogen dissemination. However, various local or infiltrating cell types have been implicated in the pathogen response but may also contribute to local tissue destruction. This has been partially abrogated clinically with the use of corticosteroids to inhibit inflammation and minimize the development of tissue pathology during many noninfectious and infectious diseases of the eye once appropriate antimicrobials have been initiated or the course is completed. This is best epitomized by the frequent need for corticosteroids in the treatment of *Toxoplasma gondii* chorioretinitis and herpetic stromal keratitis [90,164]. Systemic corticosteroids serve as broad, nonspecific immune suppressors but with many side effects. While downregulating an immune response is likely necessary to minimize long-term retinal pathology, there must be a clear identification of critical pathways in pathogen clearance and neuropathogenesis to create more specific immune modulators. To emphasize this point, an aggressive form of viral retinitis, progressive outer retinal necrosis, develops typically only in the immune-suppressed, so a broad downregulation of the immune system is not ideal. Targeted therapies will allow the clearance of the pathogen but minimize local tissue damage.

Lastly, responses within the cornea and posterior segment are similar in some respects but differ in others. Thus, findings in the cornea cannot be completely extrapolated to the posterior segment such as the retina. While this is not surprising due to the different embryological origins of ocular tissues, the diverse embryological origins, and differing responses of tissues within the eye potentially limit findings and advances from one segment to another. The inability to potentially extrapolate findings emphasizes the need for continued research in the ocular immunology, virology, and bacteriology of all segments of the eye.

## Figures and Tables

**Table 1:** Ocular infections and their common causative bacteria.

Sites of Ocular Infections and Associated Bacterial Microbes			
	Site of Infection		Associated Bacteria
Ocular Surface	Keratitis	Gram-Positive	*Staphylococcus* spp., *Enterococcus faecalis*, *Corynebacterium* spp., and *Bacillus* spp.
		Gram-Negative	*Pseudomonas aeruginosa*, *Escherichia coli*, *K. pneumoniae*, *Acinetobacter*, *Serratia marcescens*, *Serratia liquefaciens*, *Aeromonas* spp., *Fusobacterium* spp., *Enterobacter* spp., *Proteus mirabilis*, *P Pasteurella multocida*, *Morexella catarrhalis*, and *Haemophilus influenzae*
	Conjunctivitis	Gram-Positive	*Staphylococcus* spp., *Streptoccus* spp., *E. faecalis*, *Corynebacterium* spp., and *Bacillus* spp.
		Gram-Negative	*Neisseria gonorrhoeae*, *Chlamydia trachomatis*, *Escherichia coli*, *P. aeruginosa*, *Enterobacter* spp. (i.e., *E. cloacae* and *E. aerogenes*), *Citrobacter koseri*, *Proteus* spp., *Moraxella* spp., *Morexella catarrhalis*, and *Haemophilus influenzae*
	Episcleritis/Scleritis	Gram-Positive	*Staphylococcus* spp., *Streptococcus* spp., *Nocardia*
		Gram-Negative	*Pseudomonas aeruginosa*, *Klebsiella* spp., and *Mycobacterium tuberculosis*
Intraocular	Uveitis	Gram-Positive	* *Nocardia brasiliensis*
		Gram-Negative	*Mycobaterium tuberculosis*, * *Rickettsia* spp., * *Francisella tularensis*, * *Bartonella henselae*, * *Yersinia enterocolitica*, * *Pasteurella multocida*, and * *Chlamydia trachomatis*
	Endophthalmitis	Gram-Positive	*Staphylococcus* spp., *Streptoccus* spp., *Corynebacterium* spp., *Bacillus* spp., and * *Clostridium* spp.
		Gram-Negative	*Pseudomonas aeruginosa*, *Escherichia coli*, *Propiolactone* spp., *Serratia* spp., *Klebsiella pneumoniae*, *Enterobacter* spp., *Acinetobacter* spp., *Morexella catarrhailis*, and *Haemophilus* spp.
Ocular Adnexa	Dacryocystis	Gram-Positive	*Staphylococcus* spp., *Streptococcus* spp., and *Corynebacterium* spp.
		Gram-Negative	*Pseudomonas* spp., *Enterobacter* spp., *Klebsella. pneumoniae*, *Haemophilus influenzae*, *Escherichia coli*, * *Acinetobacter iwoifi*, * *Haemophilus parainfluenzae*, and * *Haemophilus aegypticus*
	Blepharitis	Gram-Positive	*Staphylococcus aureus*, *Staphylococcus* spp., *Enterococcus faecalis*, and *Corynebacterium* spp.
		Gram-Negative	*Haemophilus influenzae*

Rarely Observed *.

**Table 2:** Ocular infections and their common causative viruses, fungi, arthropods, and protozoan species.

Sites of Ocular Infections and Associated Microbes			
	Site of Infection	Infection Category	Associated Pathogens
Ocular Surface	Keratitis	Viruses	HSV, VSV, and adenovirus
		Fungi	*Candida* spp., and *Aspergillus* spp.
		Protozoa	*Acanthamoeba* spp.
	Conjunctivitis	Viruses	HSV, and adenoviruses
	Episcleritis/Scleritis	Viruses	VZV
Intraocular	Anterior Uveitis	Viruses	HSV, VZV, and CMV
	Posterior Uveitis	Viruses	HSV, VZV, and CMV
	Endophthalmitis	Protozoa	*Toxoplasma gondii*
		Viruses	
		Fungi	*Candida* spp., *Aspergillus* spp., and *Fusarium* spp.
Ocular Adnexa	Blepharitis	Mites	*Demodex folliculorum*

## Data Availability

Not applicable.
